# An apicomplexan parasite drives the collapse of the bay scallop population in New York

**DOI:** 10.1038/s41598-023-33514-3

**Published:** 2023-04-24

**Authors:** Emmanuelle Pales Espinosa, Younes Bouallegui, Denis Grouzdev, Christopher Brianik, Raymond Czaja, Sabrina Geraci-Yee, Arni Kristmundsson, Madison Muehl, Caroline Schwaner, Stephen T. Tettelbach, Harrison Tobi, Bassem Allam

**Affiliations:** 1 School of Marine and Atmospheric Sciences, Stony Brook University, Stony Brook, NY 11794 USA; 2 Institute for Experimental Pathology, University of Iceland, Keidnavegur 3, 112 Reykjavik, Iceland; 3 Marine Program, Cornell Cooperative Extension, Southold, NY 11971 USA

**Keywords:** Molecular biology, Climate sciences, Environmental sciences, Ocean sciences, Diseases

## Abstract

The bay scallop, *Argopecten irradians*, represents a commercially, culturally and ecologically important species found along the United States’ Atlantic and Gulf coasts. Since 2019, scallop populations in New York have been suffering large-scale summer mortalities resulting in 90–99% reduction in biomass of adult scallops. Preliminary investigations of these mortality events showed 100% prevalence of an apicomplexan parasite infecting kidney tissues. This study was designed to provide histological, ultrastructural and molecular characteristics of a non-described parasite, member of the newly established Marosporida clade (Apicomplexa) and provisionally named BSM (Bay Scallop Marosporida). Molecular diagnostics tools (quantitative PCR, in situ hybridization) were developed and used to monitor disease development. Results showed that BSM disrupts multiple scallop tissues including kidney, adductor muscle, gill, and gonad. Microscopy observations allowed the identification of both intracellular and extracellular stages of the parasite. Field surveys demonstrated a strong seasonal signature in disease prevalence and intensity, as severe cases and mortality increase as summer progresses. These results strongly suggest that BSM infection plays a major role in the collapse of bay scallop populations in New York. In this framework, BSM may synergistically interact with stressful environmental conditions to impair the host and lead to mortality.

## Introduction

In the last few decades, there have been an increasing number of large-scale mortality events impacting marine organisms, often associated with outbreaks of infectious diseases^1^. These events can have profound impacts on population-scale interactions, leading to degradation of ecosystem functions^2^. The causes for the increase in frequency and severity of these events are diverse but are often linked to altered environmental conditions associated with large-scale climate alterations^3^. In this framework, the outlook for the impact of disease events on marine ecosystems over the next few decades is grim.

Bivalve mollusks are one group of ecologically important marine animals that has suffered severe alterations caused by diseases^2,4^. In coastal environments, bivalves are among the most important members of benthic communities, for their ecosystem services and commercial value. Among these species, the bay scallop, *Argopecten irradians*, represents a commercially, culturally and ecologically important species found along the United States’ Atlantic and Gulf coasts. Typical habitat of adult bay scallops is mostly estuarine in shallow flats of mud, sand, and submerged aquatic vegetation with slow currents^5^. Unlike most other bivalves, bay scallops also have the ability to swim, by ‘clapping’ their two valves and shooting jets of water, to propel themselves away from predators. Adult bay scallops tolerate a wide range of temperature (6 °C to 35 °C) and salinity (12 to 35 psu), although they require warm water (20 °C and 30 °C) and high salinities (20 and 30 psu) for growth^5–7^. The bay scallop is a short-lived species that reaches maturity after one year, spawns and usually dies during their second winter. Therefore, unsuccessful recruitment in a single year can cause dramatic population fluctuations – as evidenced in highly variable commercial fishery landings throughout their range^8^. The fertilization occurs in the water column and after 14–20 days of free-swimming larval stages, the bay scallop metamorphoses and settles. The juveniles prefer to be suspended off the bottom (e.g., attached to seagrass) until they reach 20–30 mm (corresponding to 3–4 months) and then drop to the sea floor. The simultaneous hermaphrodite adult bay scallop can reach up to 70 mm and spawn from spring to fall depending on environmental factors, including temperature^5^.The major fishing area for bay scallops occurs from Massachusetts through Long Island (New York) to North Carolina^8^. The northern subspecies, *A.* *irradians irradians*, naturally present from Massachusetts to New Jersey^9^, is a major fishery resource and an emblematic species in New York where it represents the official state shell. Historically, commercial landings of bay scallops in New York started as early as 1859 when the bivalve first appeared on markets as an edible food^10^. The fishery flourished until a first adverse event in the 1930’s, when eelgrass wasting disease devastated the scallop’s preferred habitat and scallop populations declined dramatically. New York populations of *A.* *irradians* were able to recover but starting in 1985, a series of harmful algal blooms (HABs; caused by *Aureococcus anophagefferens*) occurred in New York waters, and decimated the bay scallop population^11,12^. As a direct consequence, bay scallop landings crashed from an annual average of 300,000 lbs before brown tide to 300 lbs in 1987–1988. In response to this crash, restoration efforts were initiated in 1986. The scallop populations rebounded until a severe brown tide bloom occurred in 1995, which again nearly extirpated populations^11^. A new round of restoration was begun by Cornell Cooperative Extension in 2006, with the first evidence of increased larval and juvenile recruitment in 2008; commercial landings increased as well, providing benefits to baymen and the local economy^13,14^. By 2017 and 2018, Peconic Bay landings exceeded 108,000 lbs, with an annual dockside value of $1.6 million^15^; with economic multipliers^14^, the cumulative economic benefit of the resurgence in bay scallop landings from 2008 to 2018 was over $50 million above pre-restoration baseline levels. Since 2019, however, catastrophic and recurrent summer mortality of adult bay scallops (> 90% reduction in adult biomass) have occurred annually throughout the Peconic Bays, decimating the fishery^15^ (Tettelbach et al., unpublished data). These events triggered the U.S. Department of Commerce to declare the New York bay scallop fishery a federal disaster. These mortality events were not, this time, associated with HABs as it was the case in the 1980’s. Multiple hypotheses were suggested to explain these mortalities, including synergistic interactions between intrinsic (e.g., reproductive cycle, which is known to induce stress in bivalves^16^) and extrinsic (e.g., high temperature, low dissolved oxygen, presence of pathogens) factors.

In response to the initial mortality event reported during summer of 2019, samples of scallops were collected by Cornell Cooperative Extension and the New York State Department of Environmental Conservation and submitted to Stony Brook University’s Marine Animal Disease Laboratory in November 2019. Preliminary histopathological analyses showed 100% prevalence of an apicomplexan-like parasite infecting kidney tissues (Allam, unpublished).

Following this first identification, sampling campaigns have been conducted in various field sites throughout the Peconic Bays since 2020. Multiple biological and environmental samples were secured to further explore the nature of this parasite and evaluate its impact on the scallop host. Results generated from these investigations will be reported in a suite of manuscripts including the present one. The main goal of this first manuscript is to describe histological, ultrastructural and molecular characteristics of the parasite, which was suggested to represent a non-described member of the Marosporida (Apicomplexa) and provisionally named BSM (Bay Scallop Marosporida). Molecular diagnostics tools were developed and used to monitor parasite presence and intensity in scallop tissues. The dynamics of the parasite in a representative site in the Peconic Bays is also presented as a case study. Results are discussed with a particular consideration of the potential role of climate change in the emergence of this disease and the collapse of the bay scallop population in New York.

## Material and methods

### Scallop collection and sample preparation

**Figure 1:**
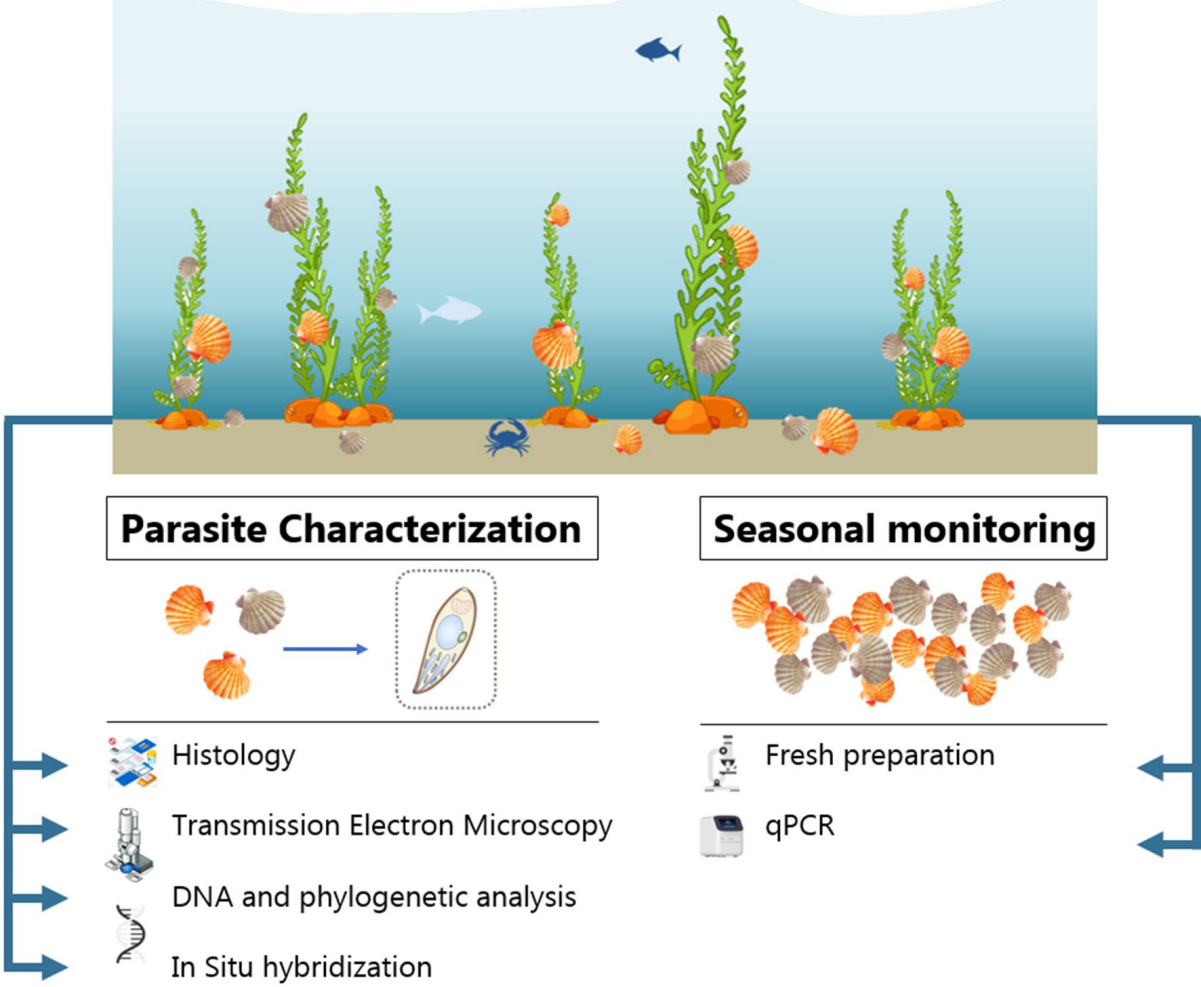
Schematic representation of the experimental workflow for parasite (Bay Scallop Marosporida) characterization and seasonal monitoring.

**Figure 2:**
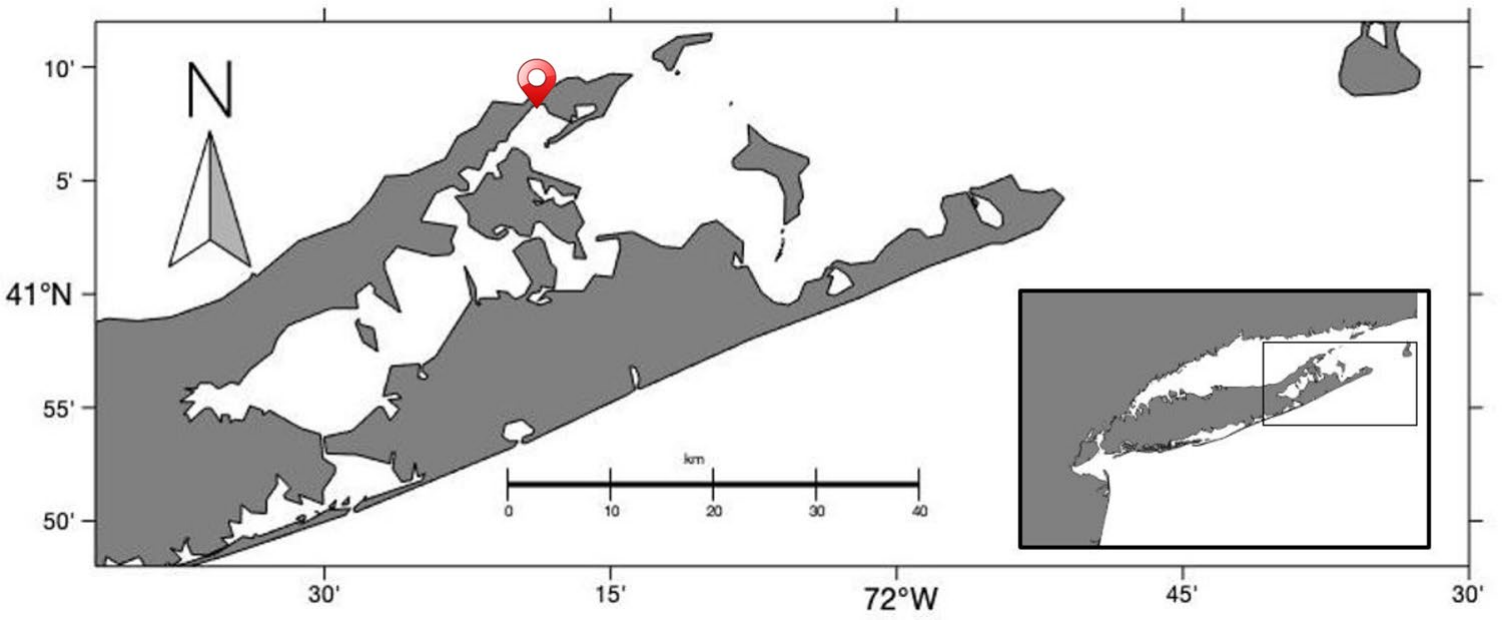
Peconic Bay in Long Island, New York. The location icon indicates the Orient Harbor sampling site.

The experimental workflow is summarized in Fig. 1. Scallops (20 to 30 adults per sampling point, for a total of 5 sampling points) were collected from Orient Harbor, NY (N 41° 08.004’, W 72° 19.393’, Fig. 2) between May and August 2021 to track BSM prevalence and severity of the infection. Scallops were cleaned, measured and dissected for multiple analysis (e.g., histological evaluation, fresh kidney preparation and DNA analysis). First, ~ 5 mm thickness cross-sections of the digestive gland, gonad, muscle, and gills were collected as well as one kidney, placed into Davidson’s fixative (1.2 L filtrated seawater, 1.2 L ethanol 95%, 0.8 L formaldehyde 36–40%, 0.4 L glycerin, 0.36 L glacial acetic acid) for one week and processed for histological evaluation using standard methods. Briefly, fixed tissues were dehydrated in an ascending ethanol series, embedded in paraffin and sectioned (5 µm thickness). One section per scallop was stained with hematoxylin and eosin, and additional sections were used for in situ hybridization (detailed below). In parallel, one small piece of the second kidney (about 25 mg) was collected, placed in 24 well microplates with 1 ml sterile artificial seawater and crushed using a plastic pellet pestle. The fresh preparation was then observed using an inverted microscope (the whole well screened using a 10 × objective and a 10 × eye piece) and parasite intensity was ranked on a scale ranging from 0 to 3 (BSM score, 0.5 incrementation) based on the percent of the surface of the preparation occupied by BSM cells as compared to scallop host cells: Stage 0 absence of the parasite (only scallop cells are visible), Stage 0.5: < 1%; Stage 1: 1% to 5%; Stage 1.5: 5 to 10%; Stage 2: 10 to 20%; Stage 2.5: 20 to 30%; and Stage 3: > 30%. Finally, the remainder of the second kidney was dissected and frozen at − 80 °C until DNA extraction.

### Transmission electron microscopy (TEM)

Kidney samples from a subset of individuals (total of 6 scallops) confirmed to be heavily infected using the fresh preparation test (described above) were used for TEM investigations. About two cubic mm from each scallop kidney were fixed in 4% glutaraldehyde in 0.2 M cacodylate buffer (pH 7.4 adjusted to 1100 mOsmol/L with sucrose and NaCl) for 6 h at 4 °C. After fixation, tissues were washed overnight in 0.2 M cacodylate buffer containing graded concentration of sucrose. Samples were then postfixed in 1% osmium tetroxide in the same buffer for 1 h at 4 °C, rinsed with 0.2 M cacodylate buffer, dehydrated through a graded ethanol series and embedded in Epon 812. Polymerization was carried out at 60 °C for 24 h. Ultrathin serial sections were cut with an ultramicrotome (Leica EM UC7/ FC7) and were mounted on copper grids. After staining with 2% uranyl acetate for 10 min and 2% lead citrate for 3 min, the grids were examined with a JEOL 1400 transmission electron microscope.

### DNA extraction and amplification

High molecular weight genomic DNA used for PacBio (Sequel II) was generated from a heavily infected kidney following a standard phenol–chloroform-isoamyl alcohol (PCI 25:24:1) protocol and ethanol precipitation. DNA quality was checked by electrophoresis on 1.5% agarose gels and DNA concentration was measured using Qubit® 3.0 fluorometer (Life Technologies, Waltham, MA). In addition, total DNA used for qPCR (quantitative polymerase chain reaction), was extracted from aliquots of kidney tissues (~ 25 mg) with the Nucleospin Tissue kit (Macherey–Nagel Inc., Allentown, PA) following manufacturer’s recommendations. DNA quality and quantity were evaluated using Nanodrop ND-1000 spectrophotometry (Thermo Scientific, Wilmington, DE).

Due to it high intraspecies variability, the internal transcribed spacer (ITS) region of the scallop parasite was chosen as the target sequence for the qPCR method^17^. To identify the BSM ITS, public apicomplexan nucleotide databases available at the National Center for Biotechnology Information (NCBI, Bethesda, MD) were screened for *18S* *rRNA* and *28S* *rRNA* sequences. These sequences (16 apicomplexan sequences, Supplementary data 1, part A) were aligned and conserved area were used to design two pairs of Apicomplexa primers (18S_BSM_F and 28S_BSM_R, Supplementary data 1) flanking the ITS target. Templates (DNA from scallops) were then amplified to generate products for cloning following the general protocols described in Pales Espinosa et al.^18^. Briefly, the PCR reaction was carried out in an Eppendorf Mastercycler (ep gradient S) using GoTaq DNA Polymerase (Promega, Madison, WI) for 10 min of initial denaturation at 95 °C, followed by 35 cycles of denaturation (95 °C, 30 s), annealing (60 °C, 30 s), and extension (72 °C, 90 s), with an additional 10 min primer extension after the final cycle. The *18S* *rRNA* sequence from the scallop parasite was generated using the primers (SFC-340f. and SFC-1260r, Supplementary data 1) and a slightly modified PCR protocol described in Kristmundsson et al.^19^. Briefly, the PCR reactions started with a 5 min initial denaturation at 95 °C, followed by 40 cycles of denaturation (95 °C, 60 s), annealing (55 °C, 60 s), and extension (72 °C, 60 s), with an additional 10 min primer extension. PCR products (e.g., ITS and *18S* *rRNA*) were run on an agarose gel electrophoresis (1.5%). The unique band (about 1500 bp and 825 bp, respectively) was cut, cleaned using the Nucleospin gel and PCR clean up kit (Macherey–Nagel Inc., Allentown, PA) and sequenced using standard Sanger sequencing.

### *18S rRNA* phylogenetic analysis

The phylogenetic position of BSM was determined using *18S* *rRNA* gene sequences of 168 representative members of the Superphylum Alveolata obtained from NCBI, including the BSM sequence. The *18S* *rRNA* gene sequences were aligned by MUSCLE^20^, and the maximum-likelihood tree was constructed with a GTR + F + I + G4 model recommended by ModelFinder^21^ in IQ-TREE^22^. Branch supports were obtained with 10,000 ultrafast bootstraps^23^. Maximum parsimony and neighbor-joining trees were reconstructed with MPBoot^24^ and MEGA11^25^, respectively.

### Apicoplast genome assembly and analysis

Purified high molecular weight gDNA was prepared for PacBio single-molecule real-time (SMRT) sequencing using the Express Template Preparation Kit 2.0 (Pacific Biosciences) and following the manufacturer’s instructions. Briefly, 2 μg of gDNA was sheared to generate 10 kb libraries using Covaris g-TUBEs and then concentrated with 0.45X AMPure PB beads (Pacific Biosciences). The sheared gDNA was enzymatically treated to remove single-strand overhangs and repair nicked DNA templates, followed by an End Repair and A-tailing reaction to repair blunt ends and polyadenylate each template. Next, overhang SMRTbell adapters were ligated onto each template and purified using 0.45X AMPure PB beads to remove small fragments and excess reagents. The purified SMRTbell libraries were then size selected at 6–50 kb using the BluePippin system on 0.75% agarose cassettes and S1 ladder, as specified by the manufacturer (Sage Science). The final size-selected library was then annealed to sequencing primer v4 and bound to sequencing polymerase 1.0 before being sequenced on one 8 M SMRTcells on the Sequel II system, producing a 20-h movie. The obtained reads were assembled using Canu v. 2.1^26^. The resulting genomic assembly was screened for the BSM apicoplast genome using BLASTn and publicly available apicoplast genomes. As a result, one scaffold was identified. PacBio reads were mapped to this scaffold using minimap2 v. 2.15-r905^27^ and used to polish the apicoplast genome with Circulator v. 1.5.5^28^. The complete apicoplast genome of BSM was 18,520 bp, with an overall G + C content of 13.5%. The apicoplast genome of BSM was annotated using Geneious^29^, and Prokka^30^. Proteins from apicoplast genomes of *Apicomplexa* were independently aligned using MUSCLE^20^, curated with Gblocks v. 0.91b^31^ with an option that allows gap positions within the final blocks, and then concatenated. The resulting alignment consists of 22 genes (*rpoB*, *rpoC1*, *rpoC2*, *rpl2*, *rpl4*, *rpl6*, *rpl11 rpl14*, *rpl16*, *rpl36*, *rps2*, *rps3*, *rps4*, *rps5*, *rps7*, *rps8*, *rps11*, *rps17*, *rps19*, *clpC*, *sufB*, *tufa*) spanning 4,485 amino acid positions. The maximum likelihood tree was calculated using IQ-TREE^22^, based on the ModelFinder recommendations^21^, and the branching support was estimated using UFBoot2^23^. MPBoot^24^ and MEGA11^25^ were used to reconstruct the maximum parsimony and neighbor-joining trees, respectively.

### Quantitative real-time PCR

The relative quantity of the parasite in scallop kidneys was determined using qPCR. Sequence information generated from the standard PCR (described above) was used to design specific primers targeting the ITS region of the scallop parasite (BSM_ITS250_F and BSM_ITS464_R, 237 bp, Supplementary data 1). Each 10 μl reaction mixture contained Takyon Low Rox SYBR 2X MasterMix blue dTTP (Eurogentec, Fremont, CA), 4.5% glycerol solution, 200 nM of each primer, and 3 μl of 1:10 diluted DNA template. Assays were carried out in triplicate in a QuantStudio 6 Flex Real-Time PCR System (Applied Biosystems) as follows: initial enzyme activation at 95 °C for 3 min, followed by 35 cycles of denaturation (95 °C, 30 s), annealing (62 °C, 20 s), and extension (72 °C, 20 s). A melting curve was generated at the end of each assay for quality control. The quantification of the parasite (correlation between the ITS region copy number to the Cq value) was determined using a standard curve built using an edited plasmid (pGEM-T Easy vector, Promega, Madison, WI) containing the ITS region amplified by the qPCR primers and cloned into JM109 competent cells (Promega, Madison, WI) following standard procedures described in Liu et al.^17^. The molecular weight of the modified pGEM-T was determined to be 2.11 × 10^15^ ng leading to the calculation that 10^9^ copies = 3.50 ng μl^−1^. To ensure optimal amplification, the circular plasmid was linearized using the restriction enzyme *Xmn*I (New England Biolabs, Ipswich, MA) and gel-purified using the Wizard Plus SV Miniprep DNA Purification and PCR Clean-Up Systems (Promega, Madison, WI). Linearized plasmid was then quantified (Qubit 3.0 Fluorometer, Thermo Fisher Scientific, Waltham, MA) to determine the plasmid copy number (see conversion relationship above) and used to build a standard curve consisting of serial dilutions ranging from 3 × 10^1^ to 3 × 10^6^ copies. The qPCR method was optimized until the characteristics of the standard curve fitted qPCR standard guidelines^32^ (amplification efficiency (E) = 100 ± 10% and the coefficient of correlation (R^2^) > 0.95). A polyserial correlation analysis was performed (polycor package in R programming) to evaluate the relationship between qPCR values (quantitative variable) and BSM scores obtained for the fresh preparation method (ordinal variable).

### In situ hybridization (ISH)

Two ISH protocols were used in this study to localize and detect the specific forms of the parasite. The first protocol was directly used as described by Kristmundsson and Freeman^33^ while the second protocol implemented slight modifications as follow. Briefly, histological sections, 5 µm thick, were deparaffinized in xylene, rehydrated in a descending ethanol series and equilibrated in phosphate buffered saline (PBS, pH 7.4). The sections were then permeabilized with 50 µg/mL proteinase K in PBS for 5 min at 37 °C followed by 2 × 5 min washes in PBS. Samples were then post-fixed in 0.4% paraformaldehyde in PBS for 15 min and subsequently washed for 2 × 5 min in distilled water. Sections were then immersed in 10% H_2_O_2_ in methanol for 10 min to prevent non-specific binding and washed in distilled water for 2 × 5 min. To omit the time-consuming pre-hybridization step, sections were dried at 45 °C for 10 min. Thereafter, sections were enclosed with Gene Frames chambers (Thermo Scientific, Boston, MA) and equilibrated in hybridization buffer made of 4SSC (saline-sodium citrate buffer) in TBS (Tri buffer saline, pH 8), containing 100 µg/mL calf-thymus DNA, 0.5% Ficoll, 0.5% polyvinylpyrrolidone, 0.5% bovine serum albumin, and 2.5 ng/µL of each of three 5′ biotin labeled oligonucleotide probes (Supplementary data 1). The 3 probes, designed by M. Freeman and A. Kristmundsson (unpublished) using the *18S* *rRNA* of *Pseudoklossia pectinis* and *Margolisiella islandica*, match the *18S* *rRNA* of the BSM and were consequently not modified. The sections were sealed, denatured at 95 °C for 4 min followed by a 90 min hybridization at 45 °C. Hybridization was followed by non-stringent and stringent washes with 2 × SSC and SSC with 0.1% Tween 20 at 42 °C, respectively. Signal detection was performed using 1/100 in PBS Streptavidin–Horseradish Peroxidase (BioLegend Incorporated, San Diego, CA) for 20 min at room temperature followed by 3 × 5 min washing in PBS and visualized with a DAB Substrate Kit (Thermo Scientific Pierce, Boston, MA). Slides were finally counter-stained with 0.5% methyl green solution or Nuclear fast red and sections were dehydrated and mounted in VectaMount (Vector Laboratories, Burlingame, CA).

## Results

### Histology and wet mounts

**Figure 3:**
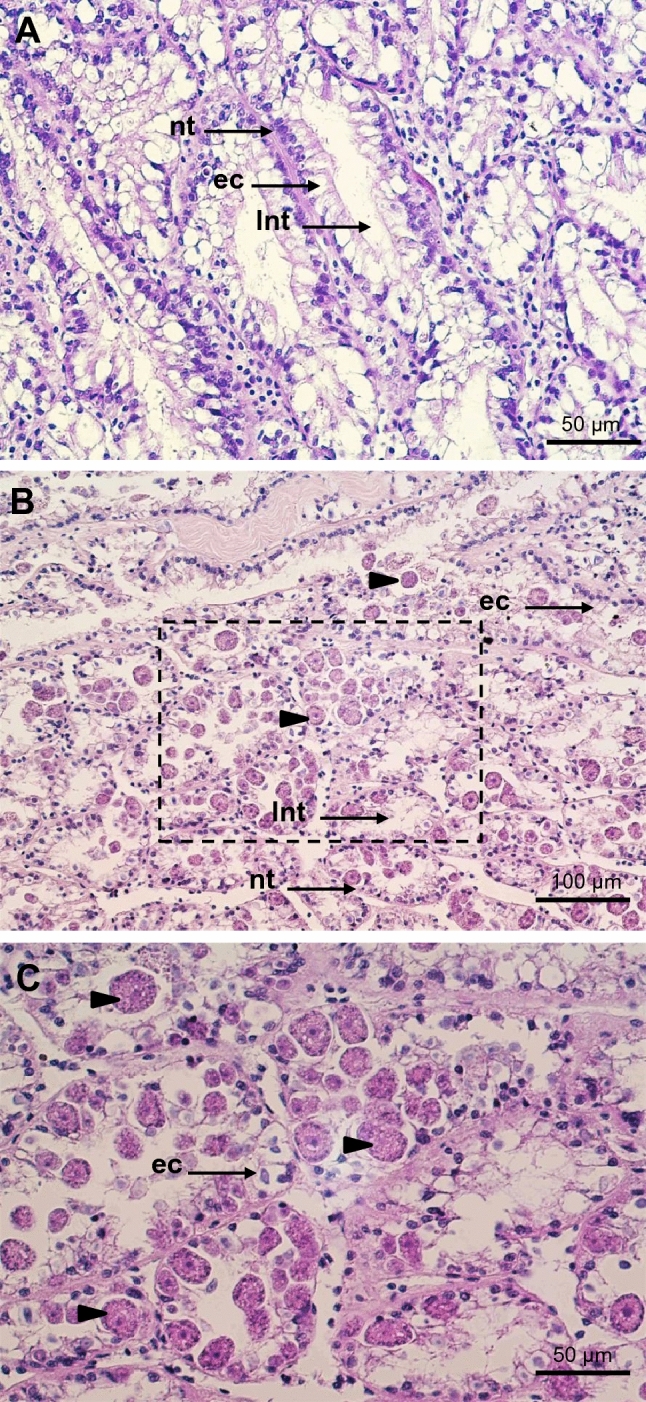
Micrographs of histological sections presenting healthy (**A**) and infected (**B** and **C**) scallop kidneys (H&E stain). (**C**) represents a high magnification capture of the area pointed in B and underlines the extensive disruption of kidney tubules caused by heavy BSM (Bay Scallop Marosporida, black arrowheads) infection. ec: epithelial cell, lnt: lumen of nephridial tubule, nt: nephridial tubule.

**Figure 4:**
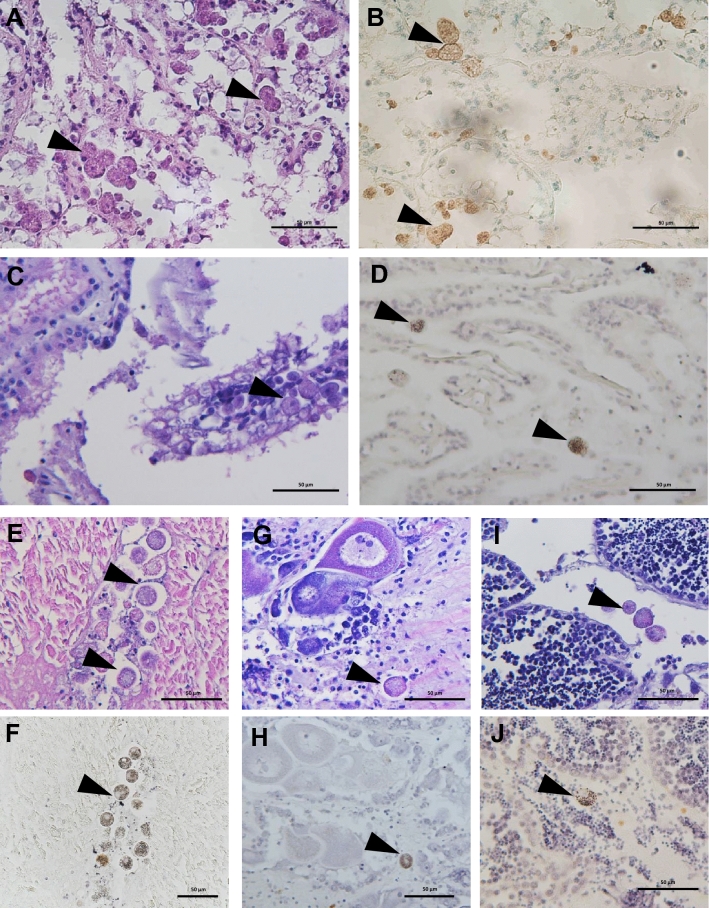
Micrographs of histological sections presenting BSM infection in different scallop tissues using H&E stain (**A**, **C**, **E**, **G** and **I**) and in situ hybridization (**B**, **D**, **F**, **H**, and **J**). (**A**) and (**B**): kidney, (**C**) and (**D**): gills, (**E**) and (**F**): adductor muscle, (**G**) and (**H**): ovary, (**I**) and (**J**): testis. The black arrowheads indicate parasite cells.

**Figure 5:**
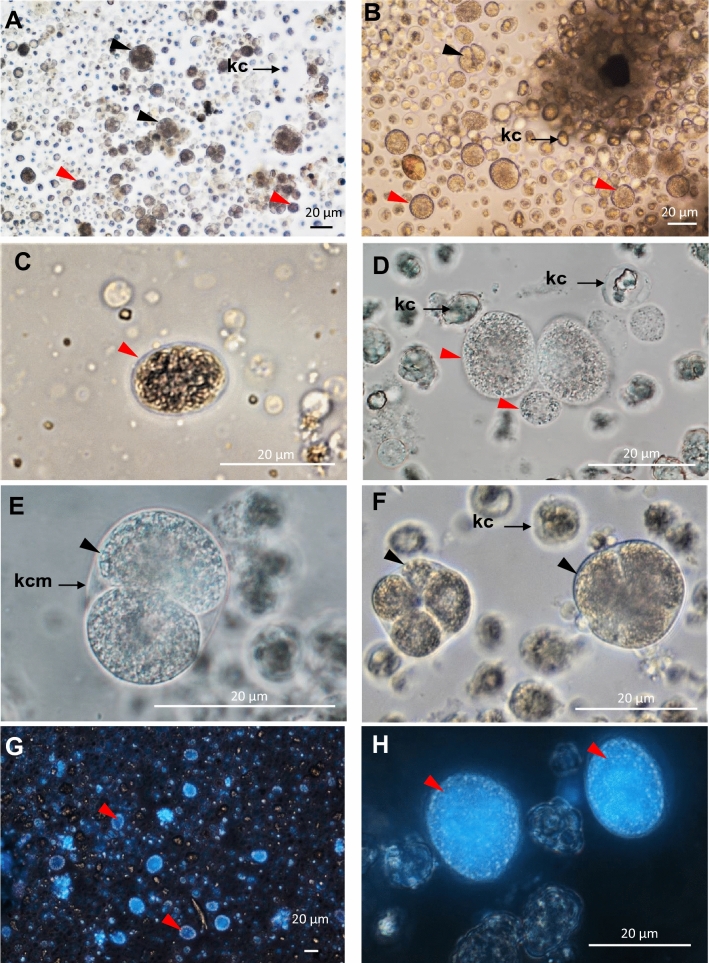
Micrographs of unstained (**A** to **F**) and stained (with DAPI, **G** and **H**) fresh kidney preparations. Red and black arrowheads represent single and dividing BSM, respectively. Letters indicate kidney cell membrane (kcm), kidney concretions (kc).

**Figure 6:**
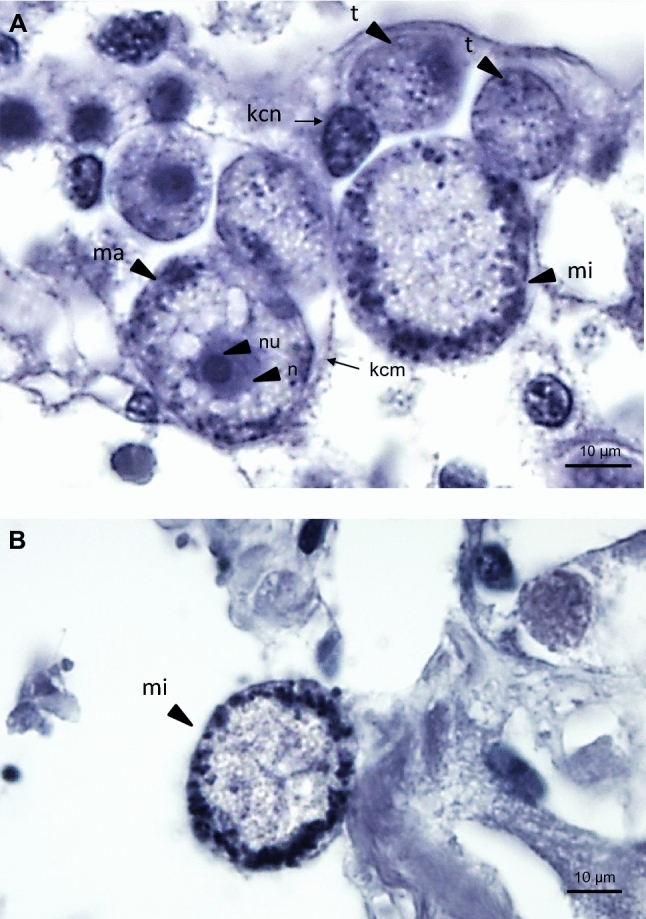
Micrographs of the most common BSM forms (**A**) found in histological section of bay scallop kidney (H&E stain). (**B**) Microgamont. Letters indicate kidney cell nucleus (kcn), kidney cell membrane (kcm), macrogamont (ma), macrogamont nucleus (n), macrogamont nucleolus (nu), microgamont (mi), trophozoites (t).

The examination of histological sections revealed that the apicomplexan parasite was most typically found in the scallop kidney, which appears to be a main target organ for BSM. Various developmental stages were observed, the majority being young growing trophozoites and macrogamonts. Severely infected kidney tissues showed heavy lesions with significant areas of the kidney displaying pathological condition, including distortion or loss of renal tubule integrity and detachment or destruction of the tubular epithelium (Fig. 3), likely leading to severe impairment of renal functionality. No notable hemocyte infiltration was observed in association with the infection. In many cases, in situ hybridization detected the parasite in other tissues, including the adductor muscle, digestive tissues (very rare), gills and gonads, associated with tissue lesions and necrosis (Fig. 4). Although some parasite forms have been found free in infected tissues (e.g., lumen of kidney tubules, between muscle fibers, connective tissue in the digestive organs), most parasite cells were found in parasitophorous vacuoles within the scallop kidney cells. The specific location of the parasite within host cells, strongly support a partial intracellular life cycle, an obligate characteristic of the Apicomplexa. This apicomplexan parasite has usually a spheroidal to ellipsoidal form and, in fresh preparations, its size varies from 5 to 30 µm, depending on life stage and maturity (Fig. 5). Both asexual and sexual forms have been observed in the samples (Fig. 6). Young trophozoites are around the low end of the size range (5–10 µm) and present an ellipsoidal form (Fig. 6). Macrogamonts were by far the most common form detected. They were either found intracellular within the renal tubular cells, but also free in the inter-tubular area, presumably after bursting of the epithelial cells. They have a spheroidal to ellipsoidal form and mature gamonts can measure up to 30 µm. Most of the gamonts present a large central nucleus and nucleolus, and a coarsely granular cytoplasm organized at the periphery of the cell (Fig. 6). Some gamonts lacked a visible nucleus suggesting they may represent microgamonts (Fig. 6), even though macro- and microgamonts are difficult to differentiate in terms of size and shape. It is noteworthy that in addition to the apicomplexan parasite, kidney cells contained abundant kidney concretions (Fig. 5).

### Ultrastructure

**Figure 7:**
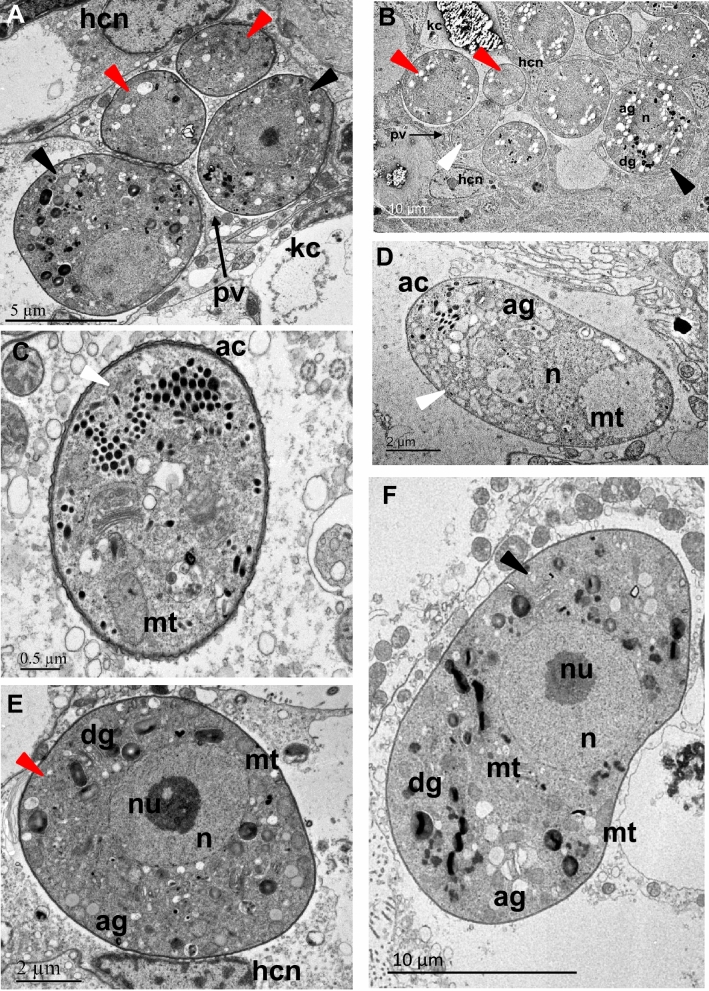
Ultrastructure of the most common BSM forms found in scallop kidney. (**A**) and (**B**): general views of kidney cells infected with multiple parasite cells. Different presumptive life stages are visible: early trophozoites (white arrowheads), growing trophozoites (red arrowheads) and gamonts (black arrowheads); (**C**) and (**D**): early trophozoites present an ellipsoidal form, contain a large mitochondrion (mt) posterior to the nucleus (n), and remnant components of the apical complex (ac), all hallmarks of the infectious sporozoite life stage that precedes the growing trophozoite stage; (**E**): a growing trophozoite presenting a round form and containing multiple vacuoles; (**F**): details of a macrogamont (sexual form) containing a large nucleus (n), several mitochondria (mt) and multiple granules. ag: amylopectin granule; dg: dense granule, hcn: host cell nucleus, kc: kidney concretion, pv: parasitophorous vacuole, nu: nucleolus.

**Figure 8:**
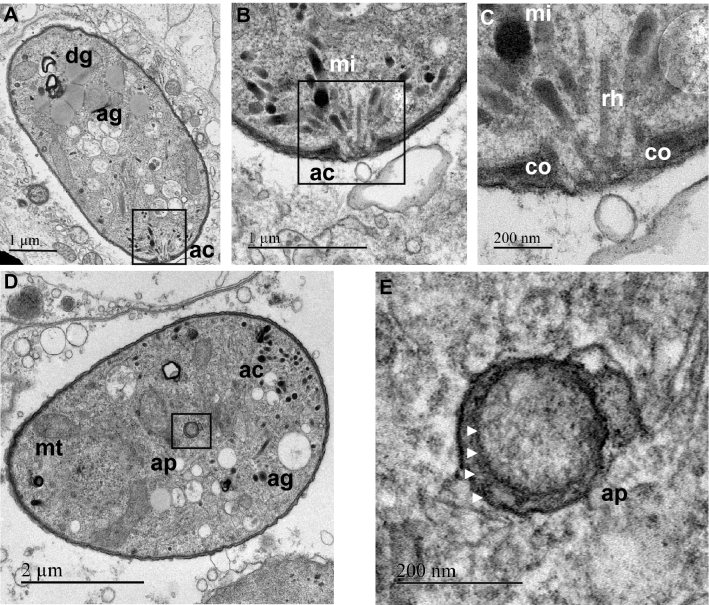
Ultrastructure of the BSM apical complex (**A** to **C**) and apicoplast (**D** and **E**). (**A**): general view of an early trophozoite with a visible apical complex (ac). (**B**) and (**C**) represent enlargement of the boxed areas in (**A**) and (**B**), respectively. Note the presence of micronemes (mi), rhoptries and conoids (co). (**D**): general view of an early trophozoite presenting an apicoplast (ap). (**E**): Larger magnification of the apicoplast surrounded by four membranes pointed out by the white arrowheads. ag: amylopectin granule; dg: dense granule, mt: mitochondria.

Ultrastructural observations (Fig. 7) revealed that the small form of the parasite contained a large mitochondrion posterior to the nucleus, and remnants of the apical complex, including microneme, rhoptry and conoid structures, all hallmarks of the sporozoite/merozoite life stage that typically precedes the trophozoite stage (Fig. 8). Sexual forms, mostly macrogamonts, were also observed in the diverse samples. Macrogamonts present a large nucleus and a typical distinct nucleolus. Their cytoplasm is coarsely granular due to the numerous inclusions and granules (e.g., lipids, amylopectin, Fig. 7). It is also important to note that more than one parasite form can be present in the same parasitophorous vacuole (Fig. 7). In addition, the apicoplast, another hallmark of the phylum Apicomplexa, was observed within the BSM (Fig. 8).

### Phylogenetic relationship

**Figure 9:**
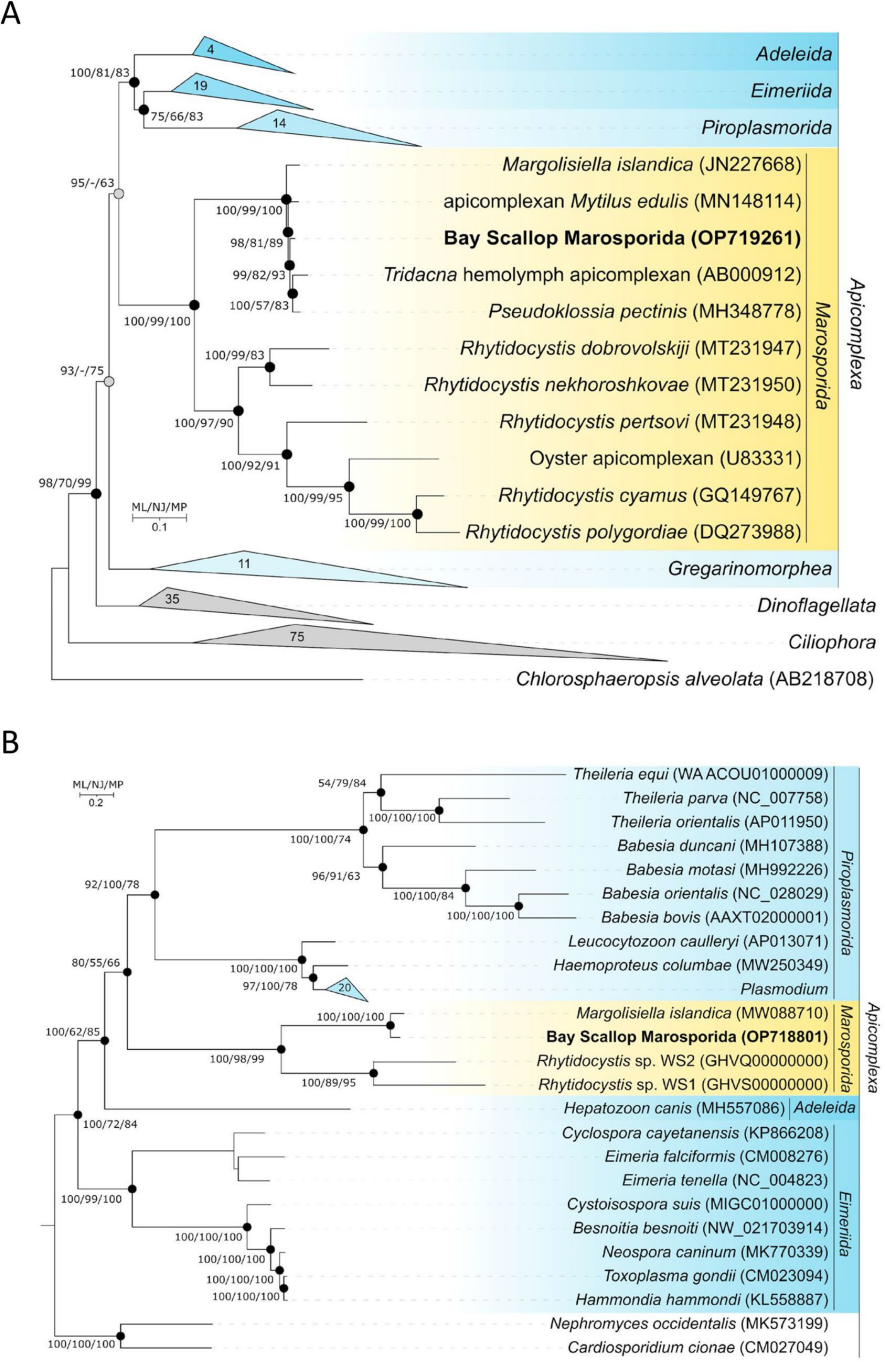
Phylogeny of the bay scallop Marosporida. (**A**). Maximum-likelihood phylogenetic tree based on *18S* *rRNA* gene sequences (1,742 nucleotide sites) reconstructed with evolutionary model GTR + F + I + G4, showing the position of BSM with closely related members of the Apicomplexa. Grey circles indicate that the corresponding nodes were recovered in the reconstructed tree based on the maximum-parsimony algorithm; black circles indicate that the corresponding nodes were recovered based on both the neighbor-joining and maximum-parsimony algorithms. Bootstrap values (> 50%) are listed as percentages at the branching points. Bar: 0.1 substitutions per nucleotide position. GenBank accession numbers for *18S* *rRNA* genes are indicated in parentheses. (**B**). The maximum-likelihood phylogenetic tree based on the amino acid alignments of 22 proteins from apicoplast genomes showing the position of BSM in relation to closely related members of the Apicomplexa. Phylogenetic analysis was performed with the LG + F + I + G4 model using 4,485 amino acid positions. Bootstrap values (> 50%) are listed as percentages at the branching points. Black circles indicate that the nodes were recovered based on both the neighbour-joining and maximum-parsimony algorithms. Bar: 0.2 amino acid substitutions per site. GenBank accession numbers for apicoplast sequences are indicated in parentheses.

The phylogenetic trees based on *18S* *rRNA* gene sequences or 22 protein sequences from apicoplast genomes show that BSM clustered with known pseudoklossids (genera *Pseudoklossia* and *Margolisiella*) and the rhydiocystids (*Rhytidocystis* spp.) that are members of the newly established Order Marosporida, including apicomplexans infecting marine invertebrates (Fig. 9A and B). For instance, BSM (OP719261, OP718801) *rRNA* clusters with *Mytilus* *edulis* apicomplexan (MN148114), *Tridacna* sp. apicomplexan (AB000912), *P.* *pectinis* (MH348778) and the closest relative *M.* *islandica* (JN227668, MW088710), described in *Chlamys islandica*^19^. Other members of this group identified in the apicoplast analysis (Fig. 9B) belong to the genus *Rhytidocystis*, which are parasitic Apicomplexa found in marine annelids^34^.

### Dynamics of the parasite in orient harbor during spring and summer 2021

**Figure 10:**
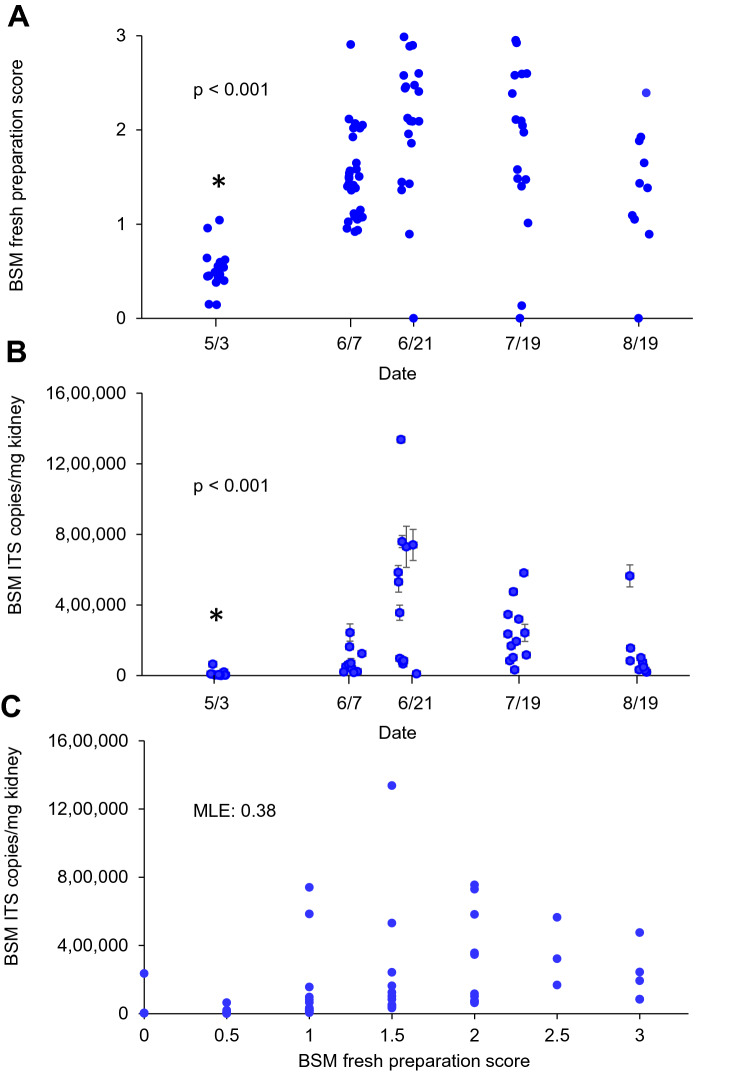
BSM infection intensity by fresh preparation score (**A**) and qPCR (**B**) in scallops collected from Orient Harbor between May 3 and August 19, 2021. Polyserial correlation between both detection techniques is presented in (**C**) where the Maximum Likelihood Estimator (MLE) is given. Asterisks indicate significant differences between May and all other sampling dates (*P*-values are given for the ANOVA on ranks in A and parametric ANOVA on Log10 transformed values in B).

The dynamics of the parasite in scallops collected from Orient Harbor in spring and summer 2021 were followed using fresh preparation and qPCR assessment of the parasite in kidney samples. On May 3, the numerical scale developed for fresh preparations (i.e., BSM score) ranged from 0 (absence of parasite) to 1 (light infection), averaging 0.41 ± 0.29 (mean ± SD, Fig. 10A), with an overall prevalence of 72.7%. Using qPCR evaluation, the average quantity of the parasite in scallop kidneys reached 11,835 ± 18,678 ITS copies/mg of kidney (Fig. 10B), with one sample below the limit of detection (100% prevalence). Scallops collected on June 7 showed a marked increase in infection intensity using the fresh preparation test (BSM scores averaging 1.59 ± 0.59, 100% prevalence) as well as by qPCR (81,606 ± 73,008 ITS copies/mg of kidney, 100% prevalence). Both diagnostic techniques showed that infection intensity continued to increase through the June 21 sampling with the fresh preparation score averaging 2.4 ± 0.54 (100% prevalence) and qPCR estimates averaging 439,594 ± 398,408 ITS copies/mg of kidney (100% prevalence). Parasite intensity tended to decrease on July 19 (2.05 ± 0.92 BSM score, 90.4% prevalence, and 241,476 ± 160,650 ITS copies/mg of kidney by qPCR, 100% prevalence) and a more marked decrease was noted on the August 19 sampling (1.62 ± 0.76 BSM score, 100% prevalence, and 123,429 ± 165,106 ITS copies/mg of kidney by qPCR, 100% prevalence). Comparisons of BSM infection intensity by qPCR (ANOVA on Log10-transformed ITS copy numbers followed by Holm-Sidak posthoc tests) and BSM scores (ANOVA on ranks followed by Dunn’s post-hoc tests) showed that disease levels were significantly lower in May as compared to all other sampling dates. Overall, results showed similar trends between both detection methods, even though the fresh preparation technique appears to underestimate infection intensity on some infections that were strongly positive by qPCR (Fig. 10C).

## Discussion

Infectious diseases have been associated with mass mortalities in mollusks worldwide^35^. The occurrence of these events is on the rise and more and more frequently associated with anthropogenic stressors and global warming^36,37^ that can exacerbate host resistance against infection, and may lead to higher pathogen virulence. The dramatic bay scallop mortality events that occur in NY every summer since 2019 are another example of this conjunction and could be the consequence of a “perfect storm” starring a parasitic infection, high energy requirements and elevated temperature.

Bivalve mollusks have been particularly impacted by mass mortality events caused by parasitic diseases. Among seven molluscan infectious diseases listed by the World Organization for Animal Health because of their economic impact, five affect bivalves, and all of these are caused by eukaryotic parasites (https://www.woah.org/). In the U.S., Dermo disease (caused by the alveolate parasite *Perkinsus marinus*) in the oyster *Crassostrea virginica* represents one of the best-case studies about the effect of climate change on bivalve health, where range extension of the disease and annual outbreaks can be directly linked to prevailing climate and seasonal weather conditions^38^. Pectinid bivalves (Family Pectinidae) are known to be susceptible to several pathogens/parasites, some of which can lead to severe damages and mass mortalities^39–41^. About 30 pathogens/infectious agents (from bacteria to arthropods) have been identified in the bay scallop *A.* *irradians*^42,43^. Results from the current study allowed the identification of an apicomplexan parasite as a main driver of the recurring mortality events in the bay scallop population in NY. Members of the phylum Apicomplexa are obligate intracellular parasites^45^ and include major pathogens of vertebrate hosts (e.g., *Plasmodium* sp., *Eimeria* sp.), while those infecting invertebrates are poorly characterized leading to difficulties in establishing accurate phylogenies^46^.

Apicomplexans are common parasites in mollusks^19,33,39,46–49^. Despite their ubiquity, their life cycles are not well known, and their systematics are frequently reexamined^50^. In bivalves, the most common apicomplexans observed are the pseudoklossids (*Pseudoklossia* spp., *Margolisiella* spp.). Most of reported species (nominal and *Pseudoklossia*-like) infect the renal tubules, but infections from other organs are also known^18^. Being obligate intracellular parasites, they often cause cellular alterations and various levels of pathological disturbances in their host. Cases of severe infections have been previously reported, where multiple organs including the digestive diverticula, gills, muscle and gonad are impacted^39,51^ (Supplementary data 2). Such heavy infections can severely affect their host (e.g., injured and necrotized tissues, inflammatory response, immunodeficiency, modification of feeding and behavior, poor health condition)^39^, leading sometimes to widespread mortality events^33,52^. For example, mass mortalities of the scallop *Chlamys islandica* in Iceland in the 2000’s were shown to result from infections caused by an apicomplexan^52^, later shown to be *Merocystis kathae* (Family Aggregatidae), a heteroxenous species having a gastropod as a definitive host^32^. The majority of apicomplexans observed in marine bivalves have been described to belong to the *Pseudoklossia*, *Margolisiella*, or *Merocystis* genera but in many cases, due to the lack of genomic information, authors are reluctant to associate observed parasites to a specific taxonomic group (Supplementary data 2). In this study, partial *18S* *rRNA* sequences suggest that BSM is a close relative to *M.* *islandica*, *P.* *pectinis* and other coccidian-like parasites found in bivalves^49^. Recently, the apicoplastal genomes and the transcriptomes of three apicomplexan parasites, including *M.* *islandica*, have been sequenced by Mathur et al.^50^. This new information was used in phylogenetic analyses, regrouping 55 taxa and covering 194 nucleus-encoded genes and 58,611 amino acid sites. Results allowed the identification of a new lineage among the Apicomplexa, designated as the Marosporida class, a sister clade of the Coccidia and Hematozoa^50^. This new class regrouped *M.* *islandica* along with the genera *Rhyditocystis*, *Merocystis* and *Aggregata*, all of which infect marine invertebrates. In our study, the analysis of partial *18S* *rRNA* sequences revealed that BSM is closely related to *M.* *islandica* and supports that BSM could represent a new member of the Marosporida class. Whole genome sequencing and assembly of the bay scallop parasite is ongoing and preliminary analysis of the BSM apicoplast genome showed a 93% similarity with the apicoplast of *M.* *islandica* (Allam et al., unpublished data). Like *M.* *islandica*^50^, the apicoplast genome of BSM is strikingly reduced (18.5 kb), with a strong AT% bias (13.5% GC). It contains the ribosomal *16S* and *23S* *rRNA* genes, as well as the plastidial genes *tufA*, *clpC*, and *sufB* (Supplementary data 3).

Apicomplexa have complex life cycles but they are all characterized by gametogonic, sporogonic and merogonic stages^53^. They can either complete their entire life cycle in one host (monoxenous, e.g., *Margolisiella* spp.) or require several hosts (heteroxenous, e.g., *Merocystis* sp.). Our preliminary results support that BSM has a monoxenous life cycle but we cannot rule out the existence of a second host. Even though some forms of the parasite have not been yet undoubtedly confirmed (e.g., oocyst, sporozoite), a hypothetical life cycle of the BSM can be proposed. The presence of an apical complex (AC) in small meronts/trophozoites, supports the existence of an initial infective sporozoite form. This AC is a unique feature of apicomplexan parasites that enables the invasion of host cells. BSM sporozoites could be acquired through the digestive system and/or the pallial organs (e.g., mantle, gills) and reach the kidneys via hemolymph, even though a direct contamination of the kidney cannot be ruled out. The young meronts/trophozoites would then undergo asexual and sexual reproduction within kidney cells. It is to note that it remains difficult to morphologically differentiate between BSM sporozoites and merozoites. In contrast, gamogonic stages were obvious in the kidney of the host and most of the observed forms share common characteristics of macrogamonts. Microgamont stages were also identified and appear to be much less abundant than macrogamonts. It should be noted that the identification of the microgamont stage (Fig. 6) was based on the absence of a discrete nucleus in the cell and what appears to be multiple DNA-rich inclusions at the periphery of the cell. Although unlikely, technical artifacts may lead to such outcomes and it is possible that BSM may have isogamous gamonts making the differentiation between microgamonts and macrogamonts difficult. This is for example the case for *P.* *pectinis*^48^, a close relative of BSM. In that species, Leger and Duboscq^48^ described the presence of multiple couples of joined gamonts, sometimes of the same size, suggesting that *P.* *pectinis* might undergo syzygy (i.e., association of gamonts prior to gametocyst formation, gamete maturation and fertilization), even though the authors did not identify other steps of the syzygy process. After fertilization, the oocysts could be released into the environment via urine. The number and the form of sporozoites released from coccidian oocysts are species-dependent^54^. However, all known pseudoklossids have polysporous oocysts with the number of sporocysts ranging from 20 to > 500^19^, but such information remains unavailable for BSM.

The description of apicomplexan parasites in bivalves^55^, including pectinids^48^, is not recent and these infections are widespread and have been reported across several continents^19,46,48,56^ (Supplementary data 2). In the Northeast of North America, apicomplexan parasites affecting bay scallops have been observed from Canada to New York^39,42,51^. In their 12-year (1972–1984) survey, Leibovitz et al.^39^ described frequent and heavy renal coccidial infections in *A.* *irradians* associated with heavy scallop mortality: *“The disease was characterized by rapid proliferation of various* *coccidial stages in renal epithelium, destruction of renal tissues and impaction of the renal tubules with tissue debris and coccidia, and continuously increasing high mortalities, often exceeding 80%”.* In 1981, Karlsson^42^ further reported that the presence of coccidia in the kidney was “common” in Rhode Island bay scallops. Therefore, parasites that are morphologically similar to BSM have been previously described in *A.* *irradians* in the region. Based on this information, it is quite reasonable to think that the BSM observed in 2019 is not a new parasite infecting scallops in the Peconic Bays of New York and it could have been described as early as 1972 by Leibovitz et al.^39^. Without genomic information from past outbreaks, however, it will be difficult to rule out any hypothesis, including possible introduction/emergence of a new, highly virulent apicomplexan species/strain.

If the prevalence of the BSM and BSM-like infections in bay scallop is usually high independently of the season, the intensity may vary from light to heavy depending on the age of the host and the season (^39,42^, this study). For example, the intensity of the infection seems to be higher in adults as compared to juveniles^39^, and higher in summer as compared to winter^42^. Light infections, characterized by the presence of a very limited number of BSM or BSM-like cells, have been found to do little harm to scallops as the structure of infected tissues remains unaltered, while the presence of heavy infections often leads to considerable kidney degradation, spread of the parasite to other organs, and eventually heavy mortalities (^39^, this study). It remains unclear whether worsening infections in the second year of scallop’s life is the result of amplification of infections acquired during the first summer and/or the outcome of a reduction in host immunity related to immune senescence in adult scallops exacerbated by stressful environmental conditions.

**Figure 11:**
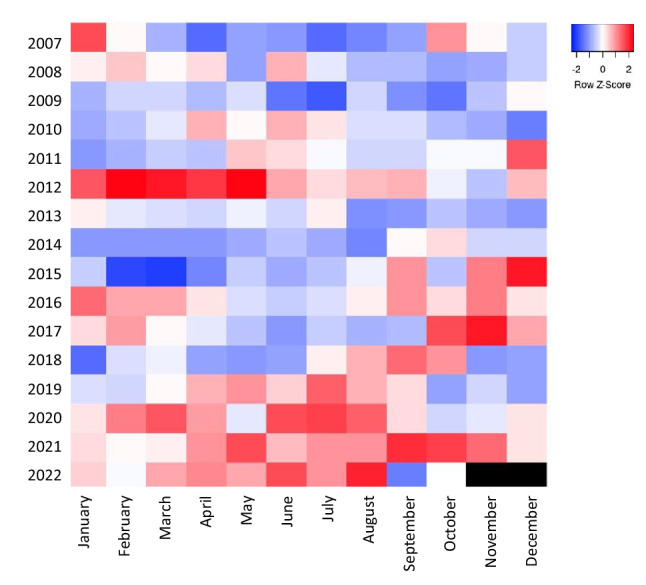
Monthly temperature anomalies calculated from 2007 to 2022 (USGS Orient Harbor Station 01,304,200). Warmer anomalies are presented in red while colder anomalies are in blue. The recurrent scallop outbreaks were first noted in summer 2019, and are systematically associated with warmer spring/summer temperatures.

Results reported here show a strong seasonal signature in disease intensity (Fig. 10). Environmental conditions during summer, and most particularly water temperature, may trigger the development of the disease, via an activation of the parasite, a reduction of host immunity, or both. Bay scallops, like many bivalve mollusks in temperate regions of the northern hemisphere, spawn primarily between late May and mid-July, with a possible second spawning event during the fall^57^. Gametogenesis and the spawning processes are critical events for marine bivalves, naturally triggered by the increase of environmental temperature and associated with a major energy requirement^58^. The conjunction between the spawning process and summer thermal stress often results in sublethal (e.g., major immunodeficiency) or lethal conditions during post-spawning periods^59^ and may have contributed to disease outbreaks and the massive scallop die-offs in the Peconic estuary since 2019. It should be noted that positive temperature anomalies (i.e., temperatures higher than the average temperature calculated from the last 15 years) were observed in the Peconic estuary during the majority of spring and summer since the occurrence of the first outbreak in 2019 (Fig. 11). Positive temperature anomalies were also noticed throughout most of 2012 and, interestingly, a large scallop mortality event was reported in the Peconic bays that summer (Tettelbach, unpublished data) although no pathology data was collected during that event. It could then be hypothesized that high temperatures may stimulate the development and proliferation of BSM, favoring infection, while increasing scallop metabolism and energy consumption. The conjunction of these factors would seriously exacerbate scallop health. It cannot be ruled out that positive temperature anomalies during winter and spring may also play a role in parasite persistence and dynamics in infected hosts as was shown in the case of *P.* *marinus* infection in the eastern oyster, where the spread and persistence of the parasite to the northeastern U.S. was directly linked to mild winters, as opposed to warm summers^60^.

## Conclusions

This study identified an apicomplexan species associated with mortality outbreaks in bay scallops, *A.* *irradians*. The parasite is closely related to the pseudoklossids (*Pseudoklossia* spp., *Margolisiella* spp.), which are members of the newly defined clade Marosporida, and is hereby dubbed bay scallop Marosporida (BSM). Diagnostic tools (rapid microscopic test and a quantitative PCR assay) were developed and used to evaluate parasite dynamics in the host. BSM infection intensity has a strong seasonal signature and is associated with an increase in scallop mortality. Heavily infected scallops display severe pathology causing extensive disruption of normal renal architecture, with likely subsequent impairment of kidney functionality. The parasite is also found in various other organs where it causes lesions and tissue necrosis. The emergence of the disease and scallop die-offs since 2019 was associated with warmer than normal springs and summers. The interplay between seasonal temperature trends, including during winter and spring, and disease dynamics, requires additional investigations. Whole genome sequencing of BSM is ongoing and is expected to enable a thorough molecular characterization of the parasite. Finally, an in-depth assessment of the mechanisms leading to mortality is needed to evaluate the contribution of direct physiological disruptions (e.g., kidney failure) vs. indirect impact (e.g., loss of fitness in infected scallops possibly leading to increased predation) of the disease on scallop mortality.

## Supplementary Information

Supplementary Information.

## Data Availability

All data generated or analyzed during this study are included in this published article and its supplementary files, or shared on NCBI. https://www.ncbi.nlm.nih.gov/search/all/?term=OP719261, https://www.ncbi.nlm.nih.gov/search/all/?term=OP718801.
