# Supplementary material for: An apicomplexan parasite drives the collapse of the bay scallop population in New York
Source: Sci Rep. 2023 Apr 24;13:6655. doi: 10.1038/s41598-023-33514-3 (PMC10126089; doi:10.1038/s41598-023-33514-3)
Supplement: Supplementary file 1 — Supplementary Information. [file 41598_2023_33514_MOESM1_ESM.pdf]

Supplementary data 1. Nucleotide sequences used to determine the ITS region of BSM (A) and build PCR primers (B). The asterisks indicate the set of primers used to amplify the 18S rRNA were designed by Kristmundsson et al., 2001. (C) *in situ* hybridization probes were designed by M. Freeman and A. Kristmundsson (unpublished) using 18S rRNA of *Pseudoklossia pectinis* and *Margolisiella islandica*. These probes perfectly match the 18S rRNA of the bay scallop Marosporida.

A

**NCBI reference**	**Species**
**18S ribosomal RNA gene**	
AF494059.1	*Adelina bambarooniae*
DQ096836.2	*Adelina grylli*
MN148114.1	Apicomplexa sp. (in mussel)
AB000912.1	Apicomplexa sp. (in tridacna)
MH590232.1	Coccidia sp.
MH567098.1	Coccidia sp.
MH590231.1	Coccidia sp.
JN227668.1	*Margolisiella islandica*
MH348778.1	*Pseudoklossia pectinis*
**28S large subunit ribosomal RNA gene**	
MH567098.1	Coccidia sp.
MH590231.1	Coccidia sp.
GU593706.1	*Eimeria papillata*
MH758782.1	*Goussia sp.*
U85705.1	*Isospora felis*
MH793412.1	*Sarcocystis fusiformis*
KR186167.1	*Sarcocystis fusiformis*
KT901264.1	*Sarcocystis hirsuta*
KJ396592.1	*Sarcocystis oviformis*

B

**Primers**	**Sequences (5' – 3")**
SFC-340f *	AGT TTC TGA CCT ATC AGC
SFC-1260r *	TCA GCC TTG CGA CCA TAC TC
18S_BSM_F	TGC CCT TTG TAC ACA CCG
28S_BSM_R	ACC ATC TTT CGT GTC CTA AC
BSM_ITS250_F	CTT GCA GCG ATG GAT CTC TTA GTT C
BSM_ITS464_R	CAA GCG TAA GCT ACA GCT CAA AAC CG

C

**ISH Probes**	**Sequences-Biotin (5' – 3")**
BSM_464r	CTG CCG CAT GGA ATA ATA CG
BSM_515r	GAA CCA AAG TAA TGA TTA ACA
BSM_725r	CAC GTT TTG ACA CTT TCA GCA

Supplementary data 2. Representative apicomplexan parasites reported in bivalve mollusks.

**Apicomplexan parasites**	**Bivalves**	**Organs**	**Location**	**References**
Hyalloklossia pelsineeri	*Donax sp.*<br>*Tellina sp.*	NA	NA	15
Margolisiella islandica	*Chlamys islandica*	Heart / hemolymph	Iceland	12
Margolisiella kabatai	*Protothaca staminea*	Kidney	British Columbia, Canada	7
Merocystis kathae	*Pectinidae and Buccinum undatum*	Muscle and kidney	Iceland	18
Pseudoklossia glomerata	*Tapes floridus*<br>*T. virgineus*	Kidney, visceral ganglia	Mediterranean Sea	16
Pseudoklossia pectinis	*Pecten maximus*	Kidney	Roscoff, France	17
Pseudoklossia pectinis-like	*Argopecten irradians*	Kidney	Rhode Island, USA	10, 11
Pseudoklossia semiluna	*Mytilus edulis*<br>*M. galloprovincialis*<br>*M. trossulus*	Kidney	British Columbia, Canada	8
Pseudoklossia (=Merocystis) tellinovum	*Tellina tenuis*	ovary	NA	2
Pseudoklossia sp.	*Argopecten irradians*	All organs	New Brunswick, Canada	4
Pseudoklossia sp.	*Argopecten irradians*	NA	Eastern Canada / Massachusetts	18, 19
Pseudoklossia sp.	*Cerastoderma edule*	kidney	Galicia, Spain	3
Pseudoklossia sp.	*M. galloprovincialis*	Kidney	Galicia, Spain	21
Indeterminate apicomplexan *(*APXSc*)*	*Aequipecten tehuelchus*	Hemocytes, gastrointestinal tract	Argentina	24
Indeterminate coccidia	*Multiple species*	Kidney	British Columbia, Canada	1
Indeterminate coccidia	*Ostrea edulis*	Kidney	Auray, France	23
Indeterminate coccidia (APX)	*Ostrea chilensis*	Connective tissue	New Zealand	22
Indeterminate coccidia	*Protothaca staminea*	Kidney	Washington, USA	20
Indeterminate coccidia	*Argopecten irradians*	Kidney, and other organs	Northeastern, USA	14
Indeterminate coccidia	*Pitar rostrata*	Kidney	Uruguay	5
Indeterminate coccidia	*Argopecten irradians*	Kidney and other organs	Prince Edward Island, Canada	25
Indeterminate coccidia	*Ensis arcuatus*	Kidney	Galicia, Spain	6
Indeterminate coccidia	*Argopecten purpuratus*	NA	Chile	9

References cited in the above table.

1 Bower, S. M., McGladdery, S. E. & Price, I. M. (1994). Synopsis of infectious diseases and parasites of commercially exploited shellfish. Ann. Rev. Fish Dis. 4, 1-199.

2 Buchanan, J. S. (1979). On two new species of coccidia from the marine bivalve, *Tellina tenuis* (Da Costa)--an adeliorine from the renal organ and an eimeriorine from the ovary. Haliotis, **8**, 57-65.

3 Carballal, M. J., Iglesias, D., Santamarina, J., Ferro-Soto, B., & Villalba, A. (2001). Parasites and pathologic conditions of the cockle *Cerastoderma edule* populations of the coast of Galicia (NW Spain). J Invertebr Pathol, **78**(2), 87-97.

4 Cawthorn, R. J., MacMillan, R. J., & McGladdery, S. E. (1992). Epidemic of *Pseudoklossia* sp./Apicomplexa) in bay scallop *Argopecten irradians* maintained in warm water recirculating facility. Fish Health Section/American Fisheries Society Newsletter, 20(2).

5 Cremonte, F., Balseiro, P., & Figueras, A. (2005). Occurrence of *Perkinsus olseni* (Protozoa: Apicomplexa) and other parasites in the venerid commercial clam *Pitar rostrata* from Uruguay, southwestern Atlantic coast. Dis Aquat Org, **64**(1), 85-90.

6 Darriba, S., Iglesias, D., Ruiz, M., Rodriguez, R., & López, C. (2010). Histological survey of symbionts and other conditions in razor clam *Ensis arcuatus* (Jeffreys, 1865) (Pharidae) of the coast of Galicia (NW Spain). J Invertebr Pathol, **104**(1), 23-30.

7 Desser, S. S. & Bower, S. M. (1997). *Margolisiella kabatai* gen. et sp. n. (Apicomplexa: Eimeriidae), a parasite of native littleneck clams, *Protothaca staminea*, from British Columbia, Canada, with a taxonomic revision of the coccidian parasites of bivalves (Mollusca: Bivalvia). Folia Parasitol. 44, 241-247.

8 Desser, S. S., Bower, S. M., & Hong, H. (1998). *Pseudoklossia semiluna* n. sp. (Apicomplexa: Aggregatidae): a coccidian parasite of the kidney of blue mussels, species of *Mytilus*, from British Columbia, Canada. Parasite, **5**(1), 17-22.

9 DiSalvo, L. (1994). Chronic infection of broodstock as a potential source of substandard gametes and larval infection in Chilean scallop hatcheries. Proceedings of the 9th International Pectinid Workshop, April 22-27, 1993 1, 107-111.

10 Karlsson, J. D. (1981). Hydrographic and biologic studies of Rhode Island coastal salt ponds. (Rhode Island Department of Environmental Management). 40pp.

11 Karlsson, J. D. Parasites of the bay scallop, *Argopecten irradians* (Lamarck, 1819). (1991). An International Compendium of Scallop Biology and Culture., 180-190.

12 Kristmundsson, Á., Helgason, S., Bambir, S. H., Eydal, M. & Freeman, M. A. (2011). Margolisiella islandica sp. nov. (Apicomplexa: Eimeridae) infecting Iceland scallop *Chlamys islandica* (Müller, 1776) in Icelandic waters. J. Invertebr. Pathol. 108, 139-146.

13 Kristmundsson, Á. & Freeman, M. A. (2018). Harmless sea snail parasite causes mass mortalities in numerous commercial scallop populations in the northern hemisphere. Sci. Rep. 8, 1-12.

14 Leibovitz, L., Schott, E. & Karney, R. (1984). Diseases of wild, captive and cultured scallops. J. World. Aquac. Soc. 15, 267-283.

15 Léger, L. (1897). Sur la présence des coccidies chez les mollusques lamellibranches. C R Seances Soc Biol Fil, **49**, 987-988.

16 Léger, L. & Duboscq, O. (1915). *Pseudoklossia glomerata* ngn sp., coccidie de lamellibranche. Arch Zool. Exp. Gén. (Suppl. Notes Rev.) 55, 7-16.

17 Léger, L. & Duboscq, O. (1917). *Pseudoklossia pectinis* n. sp. et l'origine des adéleidées. Arch. Zool. Exp. Gén. (Suppl Notes Rev.) 68, 88-94.

18 McGladdery, S. E. (1990). Shellfish parasites and disease of the east coast of Canada. Bull. Aquac. Assoc. Can, **90**, 14-18.

19 McGladdery, S. E., Bradford, B. C. & Scarratt, D. J. (1993). Investigations into the transmission of parasites of the bay scallop, *Argopecten irradians* (Lamarck, 1819), during quarantine introduction to Canadian waters. J. Shellfish Res. 12, 49-58.

20 Morado, J. F., Sparks, A. K. & Reed, S. K. A (1984). Coccidian infection of the kidney of the native littleneck clam, *Protothaca staminea*. J Invertebr Pathol, **43**, 207-217.

21 Robledo, J. A. F., Santarém, M. M., & Figueras, A. (1994). Parasite loads of rafted blue mussels (*Mytilus galloprovincialis*) in Spain with special reference to the copepod, *Mytilicola intestinalis*. Aquaculture, **127**(4), 287-302.

22 Suong, N. T., Webb, S., Banks, J., Wakeman, K. C., Lane, H., Jeffs, A., ... & Fidler, A. (2017). Partial 18S rRNA sequences of apicomplexan parasite 'X'(APX), associated with flat oysters *Ostrea chilensis* in New Zealand. Dis Aquat Org, **127**(1), 1-9.

23 Tige, G., Comps, M. & Grizel, H. (1977). Presence d'une coccidie parasite du rein chez *Ostrea edulis* L. Revue des Travaux de l'Institut des Pêches Maritimes 41, 223-225.

24 Vázquez, N., Freeman, M. A., Cremonte, F., Gilardoni, C., & Kristmundsson, Á. (2022). A phylogenetically unresolved apicomplexan (APXSc) causing swirl lesions in the Tehuelche scallop, *Aequipecten tehuelchus*, from the Southwest Atlantic coast. Int J Parasitol Parasites Wildl, **17**, 295-307.

25 Whyte, S., Cawthorn, R. J. & McGladdery, S. (1994). Co-infection of bay scallops *Argopecten irradians* with *Perkinsus karlssoni* (Apicomplexa, Perkinsea) and an unidentified coccidian parasite. Dis. Aquat. Org. 18, 53-62.

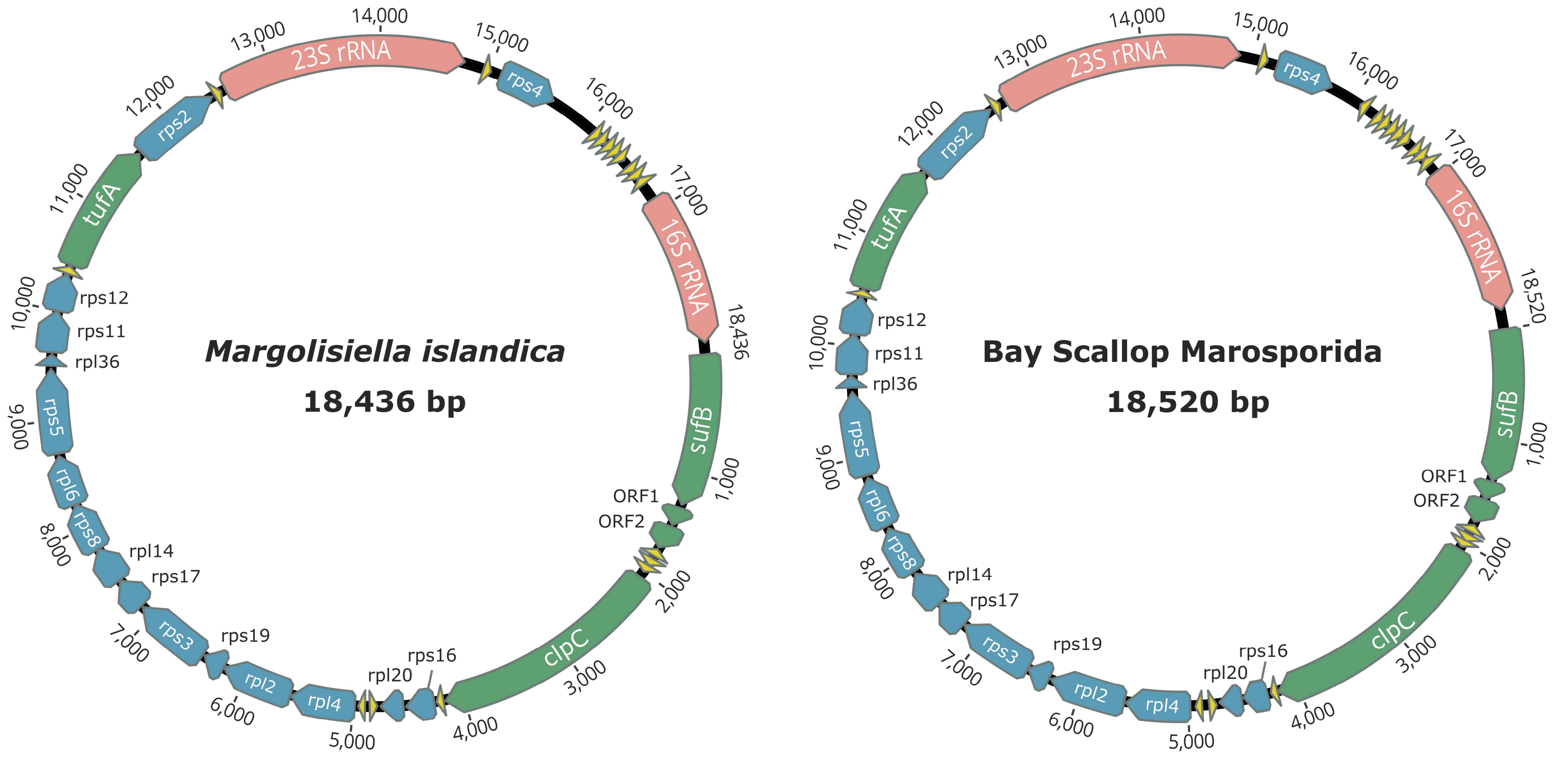

Supplementary data 3. The complete apicoplast genomes of *Margolisiella islandica* and BSM with visualization showing the ribosomal proteins (blue), other proteins (green), rRNAs (pink) and tRNAs (yellow).
